# A flexible format LAMP assay for rapid detection of Ebola virus

**DOI:** 10.1371/journal.pntd.0008496

**Published:** 2020-07-31

**Authors:** Laura C. Bonney, Robert J. Watson, Gillian S. Slack, Andrew Bosworth, Nadina I. Vasileva Wand, Roger Hewson

**Affiliations:** Public Health England, National Infection Service, Porton Down, Salisbury, Wiltshire, United Kingdom; Deakin University, AUSTRALIA

## Abstract

**Background:**

The unprecedented 2013/16 outbreak of *Zaire ebolavirus* (Ebola virus) in West Africa has highighted the need for rapid, high-throughput and POC diagnostic assays to enable timely detection and appropriate triaging of Ebola Virus Disease (EVD) patients. Ebola virus is highly infectious and prompt diagnosis and triage is crucial in preventing further spread within community and healthcare settings. Moreover, due to the ecology of Ebola virus it is important that newly developed diagnostic assays are suitable for use in both the healthcare environment and low resource rural locations.

**Methodology/Principle findings:**

A LAMP assay was successfully developed with three detection formats; a real-time intercalating dye-based assay, a real-time probe-based assay to enable multiplexing and an end-point colourimetric assay to simplify interpretation for the field. All assay formats were sensitive and specific, detecting a range of Ebola virus strains isolated in 1976–2014; with Probit analysis predicting limits of detection of 243, 290 and 75 copies/reaction respectively and no cross-detection of related strains or other viral haemorrhagic fevers (VHF’s). The assays are rapid, (as fast as 5–7.25 mins for real-time formats) and robust, detecting Ebola virus RNA in presence of minimally diluted bodily fluids. Moreover, when tested on patient samples from the 2013/16 outbreak, there were no false positives and 93–96% of all new case positives were detected, with only a failure to detect very low copy number samples.

**Conclusion/Significance:**

These are a set of robust and adaptable diagnostic solutions, which are fast, easy-to-perform-and-interpret and are suitable for use on a range of platforms including portable low-power devices. They can be readily transferred to field-laboratory settings, with no specific equipment needs and are therefore ideally placed for use in locations with limited resources.

## Author summary

This study describes the development of a set of interchangeable diagnostic assays for the detection of Ebola virus in patient samples. Each are rapid-turnaround, highly-specific (showing no detection of non-Ebola strains), sensitive and portable. These assays are ideally placed for field-use during outbreaks in low-resource countries and equally suited to use as conventional high-throughput laboratory tests. They encompass a colourimetric option with easy-to-interpret pink-to-yellow colour-change visualisation and real-time detection formats allowing the approximation of virus copy number, which helps monitoring of virus levels during infection. The inclusion of a probe-based detection method also leaves the door open for multi-pathogen detection, which could be useful for future VHF outbreaks, where the cause is unknown.

## Introduction

The recent 2013/16 outbreak of EVD in West Africa caused by the Ebola virus, Makona variant (*ebolavirus*, *Filoviridae*) resulted in an estimated 28,610 infections and approximately 11,308 deaths, with a case fatality rate of 28–67% (based on WHO figures)[1, 2]. The current outbreak in the Democratic Republic of the Congo, caused by the same *Zaire ebolavirus* species *(source MSF*, *based on data released from the national laboratory (INRB))*[3] has already 3291 confirmed/probable cases and 2193 confirmed/probable deaths as of 12^th^ November 2019 (source WHO data)[4, 5], demonstrating the continuing need for good diagnostic capability for Ebola virus. EVD is a febrile, multisystem illness, with a predominance of gastrointestinal symptoms and signs such as nausea, vomiting, diarrhoea, and abdominal pain, which frequently lead to hypovolaemia, metabolic acidosis, renal dysfunction, and multi-system organ dysfunction. Death is normally due to hypotensive shock and multiple organ failure [6–8]. Although some vaccines and interventions have shown promise, such as rVSV-ZEBOV and cAd3-ZEBOV vaccines and neutralising monoclonal antibody cocktail-based therapeutics such as ZMapp [6, 9, 10] EVD still has no reliable, proven cure. Treatment regimens generally rely on supportive care and patient management to aid natural recovery [9]. Ebola virus is a ACDP (Advisory Committee on Dangerous Pathogens) Hazard Group (HG) 4/BSL-4 (Biosafety Level-4) pathogen with a rapid transmission profile and can be contracted through direct contact with highly infectious bodily fluids such as blood, faeces and vomit [6, 11, 12]. Preventing spread from person-to-person is key to avoiding large-scale community transmission. This is particularly a problem in rural settings where healthcare and testing facilities are limited and living conditions may be overcrowded with insufficient sanitation [12]. Early diagnosis can help prevent spread, facilitating efficient implementation of infection control measures such as barrier nursing, patient isolation, contact tracing and ring vaccination. Rapid and accurate diagnosis also accelerates triage of patients in hospital settings, reducing prolonged exposure of negative suspected cases to true positives [12–14].

Ebola diagnostic technology can take several forms. Traditional methods encompass resource-heavy large scale batch testing platforms with the capability of high-throughput testing by highly trained technicians. These are typically set up in key strategic positions that are likely to be near large urban centres and major hospitals. Alternatives include rapid point-of-care (POC) diagnostics suitable for field laboratory testing, or full sample-to-answer devices for use by minimally trained operators in local community clinics or the patient bedside. Rapid diagnostics commonly involve nucleic acid detection [15] or serology[16], however some serological methods may produce false positives due to cross-reactivity [17]. Additionally, the detection of immune responses in EVD patients may be hampered by the varying ability of such individuals to mount an immune response [14].

In the rapid POC setting, some RT-PCR-based methods of nucleic acid target amplification and detection have shown promise (e.g. Cepheid GeneXpert and the BioFire film Array) [16]. The need for extraction and thermal cycling in these devices however limits speed and increases complexity, footprint, weight and power needs, reducing potential for bedside testing [15, 16]. Even for the more sophisticated micro-PCR systems which circumvent the restrictions to speed and portability, the issues of cost are potentially prohibitory in resource-poor settings [18]. The use of isothermal target amplification avoids many of the issues associated with RT-PCR by negating the need for thermal cycling. This significantly speeds-up target amplification and detection and reduces the power needs of equipment, thereby increasing portability, lowering running costs and reducing the time-to-result in conventional devices. Furthermore, isothermal assays have shown resilience to the presence of contaminants in crude unprocessed samples, with the potential to simplify the procedure and increase throughput [19–21]. Overall, the reduced time-to-detection and the use of lightweight portable detection devices, makes isothermal diagnostics ideal for use in field laboratories. Isothermal diagnostics may also have greater potential for use in miniaturised fully-automated bedside testing due to reduced instrument size and complexity [18].

There are several isothermal amplification methods potentially suited to POC diagnostics such as LAMP (loop-mediated Isothermal Amplification) and RPA (Recombinase Polymerase Amplification) [22]. LAMP has many positive attributes, combining the benefits of speed, simplicity of set-up and the potential for multiple detection outputs, with the use of a complex set of primers reducing the chances of false positives.

In this study we designed a rapid, flexible Ebola virus diagnostic that could be used as a POC assay in future Ebola virus outbreaks, with additional adaptability for multiplexing and conventional high-throughput use. We designed the diagnostic primarily for use in the POC setting, as a low throughput, rapid and simple-to-use assay for field laboratory use in resource limited settings, with potential to adapt to bedside testing. To achieve this, we developed a diagnostic with 3 possible readouts; a probe-based detection for future multiplexing, a colour change detection method for ease of result readout by minimally trained operators and a conventional intercalating dye-based method to provide a basis for comparison. Each format is suited to both the high throughput laboratory setting and a portable lightweight device, but the probe and colour change methods have additional attributes that increase their potential utility in future EVD outbreaks.

## Materials and methods

### Ethics statement

This research study was conducted using the Ebola Biobank, which is a partnership between Ministry of Health and Sanitation of Sierra Leone and Public Health England (PHE).

West African sera tested in this study were provided to the EbolaMoDRAD consortium (of which this project forms a part), as part of a biobank of samples collected during the 2013/16 outbreak from laboratories in Sierra Leone in a partnership between PHE and the Ministry of Health and Sanitation of Sierra Leone (PHE MOHS biobank). Ethical approval was obtained from both the PHE Research Ethics and Governance Group ((REGG) (reference: PHE R&D 336)) and the Sierra Leone Ethics and Scientific Review Committee (SLESRC (permission received from SLESRC 10th August 2017)). Permission was granted for use of the samples by members of the consortium to validate diagnostic assays developed as part of the project. Patient data was anonymised to remove patient identifiers and clinical information before the start of the study. In addition, UK urine and serum samples collected for the crude sample testing were provided by anonymised volunteers at PHE. The collection and use of UK human serum and urine samples for testing of diagnostic assays was approved by the PHE REGG (reference: PHE R&D 309) and written consent was obtained.

### Primer and probe design

The Ebola virus LAMP assay primers and probe were designed to the nucleoprotein (NP) gene, following alignment of a range of Ebola virus strains from 1976–2014. Several sets of primers (F3, B3, FIP, BIP, Loop F, Loop B) were designed using LAMP designer 1.10 software, with standard settings. The primer sets were tested sequentially for amplification of synthetic templates using SYBR Safe DNA gel stain and real-time detection on the Applied Biosystems 7500 (ABI 7500) real-time PCR system and a lead set selected for further validation. An assimilating probe was designed in two parts, with one strand designed to the Loop F region and a separate quencher strand (based on the chemistry described in Kabota and Jenkins 2015 and Kabota et al 2011 [23–25]).

### Primer and probe preparation

LAMP primers were provided by Integrated DNA Technologies (IDT) and probe and quencher sequences by ATDBio, all as HPLC purified material. The primers were reconstituted as 100μM stocks in pH 8.0 Tris EDTA buffer, then prepared as a primer mix in molecular grade distilled water with a concentration of 1μM F3 & B3, 2μM Loop F & Loop B and 8μM of FIP & BIP. Probe and quencher were reconstituted as 100μM stocks in pH 8.0 Tris EDTA buffer and were prepared separately in working stocks of 4μM probe and 6μM quencher in pH 8.0 Tris EDTA buffer. Primer mixes and separate probe and quencher were then frozen at -20°C in single use aliquots.

### Synthetic Ebola virus NP RNA template preparation

Synthetic 2kb Ebola virus NP DNA fragments were prepared with T7 and SP6 promoters respectively at the 5’ and 3’ ends, by IDT from a range of strains (AF086833, AY354458, KC242788, KR817135, KJ660348), (see S1 Fig and S2 Fig). Fragments were reconstituted in Tris EDTA buffer pH 8.0 (10ng/μl), then PCR amplified (2ng/reaction) to increase copy number, using a New England Biolabs (NEB) Q5 Hot Start High-Fidelity mastermix and T7 tail forward (5’GCGCTAATACGACTCACTATAGGG 3’) and SP6 tail reverse (5’GCGCATTTAGGTGACACTATAGAA 3’) primers, following the method described by Wand et al 2018 [26]. The PCR products were gel extracted using a 1% TAE agarose gel and a Qiagen QIAquick gel extraction kit. RNA fragments were generated from the DNA templates with a NEB T7 High Yield RNA synthesis kit following the kit instructions. A 0.2ml PCR tube was prepared with 2μl volumes of individual NTPs (100mM stocks of ATP, UTP, GTP and CTP), 2μl T7 RNA polymerase mix, 2μl 10X reaction buffer and sufficient dH_2_0 for a final volume of 20μl after addition of approximately 1μg template DNA. The reaction was then incubated for 2 hours at 37°C, prior to DNAse-treatment using a NEB DNase I kit; 68μl molecular grade water, 10μl 10X DNase I buffer and 2μl RNAse-free DNase I were added to each reaction, with incubation at 37°C for 15 mins. The fragments were purified using a Qiagen RNeasy mini kit as per manufacturers’ instructions, with quantification by Qubit (Thermo-Fisher) using an RNA broad-range assay kit.

### Crude sample preparation

Pools of human serum and urine samples were prepared from samples donated by healthy UK volunteers and a human saliva pool was purchased from Lee Biosolutions Inc. Bodily fluid pools were stored at -20°C in single use aliquots and diluted 1 in 10 in molecular grade distilled water prior to testing.

### Ebola virus real-time LAMP assays on the ABI 7500 Real-Time PCR platform

The real-time LAMP assays were performed in a 25μl volume using the NEB WarmStart LAMP kit. For the intercalating dye LAMP, a fresh 25X working stock of SYBR Safe DNA gel stain (Life Technologies Ltd Invitrogen Division) was prepared each time by diluting the stock 1 in 400 in molecular grade distilled water. For the intercalating dye assay, a reaction mastermix was prepared composed of the following per reaction;12.5μl Warmstart LAMP buffer, 5μl primer mix, 1.5μl molecular grade distilled water and 1μl 25X SYBR Safe stock. For the probe assay, a reaction mastermix was prepared composed of the following per reaction;12.5μl Warmstart LAMP buffer, 5μl primer mix, 1.5μl molecular grade distilled water and 0.5μl each of the probe and quencher working stocks. A 20μl volume of mastermix was distributed to the wells of a 96-well PCR plate, 5μl of template/test sample was added and the plate was then briefly centrifuged before being run on an ABI 7500 system for 35 minutes at 65°C, with fluorescence detection every 60 seconds in the FAM channel. A threshold was set at 200,000 delta Rn as this provided good sample recognition whilst avoiding the potential for background fluorescence detection. A cut-off was set for the intercalating dye assay of 25 minutes.

Where crude samples were included, 5μl of crude sample was added together with 1μl of template and 1.5μl of distilled water was omitted from and 0.5μl Invitrogen RNAseOUT (40U/μl) added to the original mastermix to provide a final volume of 25μl.

### Ebola virus real-time LAMP assays on the OptiGene Genie III portable isothermal platform

The real-time integrating dye LAMP assay was performed in a 25μl volume using the NEB WarmStart LAMP kit as described in the proceeding section, with the exception that the reactions were run in Genie 8-well tube strips on a Genie III platform, with data analysis performed using the OptiGene Genie Explorer software.

### Ebola virus colour LAMP

The colour change LAMP was performed in a 25μl volume using the NEB WarmStart colour LAMP reagent. A reaction mastermix was prepared composed of the following per reaction;12.5μl Warmstart colour LAMP reagent, 5μl primer mix and 2.5μl molecular grade distilled water. A 20μl volume of mastermix was distributed to the wells of a 96-well PCR plate, 5μl of template/test sample was added and the plate was then briefly centrifuged before being incubated on a 96 well thermal block for 35 minutes at 65°C. The plates were placed on a 96 well cold block before visualisation. Unless otherwise stated a sample was deemed positive if it appeared yellow in colour and a sample was deemed negative if it was pink in colour.

Where crude samples were included, 5μl of crude sample was added together with 1μl of template and 1.5μl of distilled water was omitted from and 0.5μl Invitrogen RNAseOUT (40U/μl) added to the original mastermix to provide a final volume of 25μl.

### Ebola virus real-time PCR

This method, chosen as the reference test as it is a PHE gold-standard RT-PCR for Ebola virus, was adapted from the work of Trombley et al 2010 [27]. Briefly, the RT-PCR assay was performed in a 20μl volume, using a Taqman Fast Virus 1-Step Master Mix. Each reaction was composed of the following; 5μl Taqman reagent, 0.9μM each of F565 and R640 primers, 0.25μM p597S probe and sufficient molecular grade distilled water to make a final volume of 20μl after addition of template. Mastermix was distributed to the wells of a 96-well PCR plate and 5μl of template was added, the plate was briefly centrifuged before being run on an ABI 7500 system using the following cycling conditions; hold for 5 mins at 50°C, hold at 95°C for 20 sec, then 45 cycles of (95°C for 3 sec, 60°C for 30 sec). Fluorescence was detected once per cycle, in the second cycling step in the FAM channel and ROX was used as a passive reference. A threshold was set at 0.1 delta Rn following the in-house PHE protocol for this published assay.

Primer and probe sequences are as described by Trombley et al 2010 [27].

### Ebola virus positive viral panel

The positive viral panel was formed from a collection of viral extracts of Ebola virus strains, cultured and extracted by members of the Virology and Pathogenesis and High Containment Microbiology groups at PHE Porton. Viral RNA was extracted using standard Qiagen RNA extraction kits. Synthetic NP viral RNA fragments spanning 2kb of the 2.2kb Ebola virus NP were used to supplement the collection.

### Negative viral panel

Viral RNA extracts from cultured reference samples covering the genera: *Orthonairovirus*, *Orthobunyavirus*, *Orthohantavirus*, *Mammarenavirus*, *Phlebovirus*, *Flavivirus*, *marburgvirus and ebolavirus* were provided by the Virology and Pathogenesis group, High containment Microbiology (HCM) group and the National Collection of Pathogenic Viruses (NCPV) at PHE Porton and were confirmed as positive using PCR assays (Pan-family and/or strain-specific). Pan-family assays included a Pan-*Orthobunyavirus* RT-PCR (primer pair A), a New World arenavirus RT-PCR (GuaS2041+, GuaS2333a-/b-/c-) and a Pan-*Orthohantavirus* RT-PCR adapted respectively from published assays [28–30] and used with the Invitrogen SuperScript III One-Step RT-PCR System, with detection on Invitrogen E-gel EX with Sybr Gold II and a Pan-*Orthonairovirus* RT-PCR (personal communication from Kurosaki Y). Also included was a Pan-*Flavivirus* RT-qPCR (Flavi all S, Flavi all S2, Flavi all AS4, Flavi all probe mix 3) adapted from a published assay [31] and used with Invitrogen Superscript III Platinum One-step *Quantitative* RT-PCR kit, with detection on an ABI 7500 system. A Lassa-specific assay was adapted from a published assay [32] and used with the Invitrogen SuperScript III One-Step RT-PCR System, with detection on Invitrogen E-gel EX with Sybr Gold II. A Rift Valley Fever virus-specific assay was adapted from a published assay [33] and used with a ABI Fast virus 1-step master mix, with detection on the Thermo Fisher ViiA 7 PCR system. An Altona RealStar Filovirus type RT-PCR kit was used to identify non-*Zaire ebolavirus*, *ebolavirus* strains.

### Preparation of clinical samples for testing on the Ebola virus LAMP

Serum samples from the 2013–2016 Ebola virus outbreak in Sierra Leone were provided by PHE and the Sierra Leone government from the PHE-MOHS biobank. Sera samples chosen for the test panel included negative sera to help determine assay specificity and positive sera with a spread of Ct values (from Ct 15.2 to Ct 34.6) to tests the limits of assay capability, with the panel size of 50 determined by availability. As such the index test was not blinded in respect to the reference test results, but this had no impact on data interpretation. Inactivation was performed by the HCM group at PHE, with inactivation using AVL buffer and ethanol; 140μl sample added to a 2ml screw-cap tube with 565.6μl of freshly prepared AVL-cRNA. This was mixed and incubated at room temperature for 10 minutes, before 560μl of ethanol was then added and the sample mixed again. Inactivated serum samples were extracted using a Qiagen QIAamp viral RNA mini kit according to manufacturer’s instructions, with elution into 120μl AVE buffer.

### Statistical analysis of clinical sample testing data

Test data was analysed using MEDCALC (Diagnostic test evaluation calculator) statistical software using the formulae described below. For the formulae below a = number of true positives detected, b = number of false negatives, c = number of false positives and d = number of true negatives.

Sensitivity = a / (a + b)

Specificity = d / (c + d)

Positive Predictive Value (PPV) = a / (a + c)

Negative Predictive Value (NPV) = d / (b + d)

Accuracy = (a + d) / (a + b + c + d)

## Results

### Assay design

**Fig 1:**
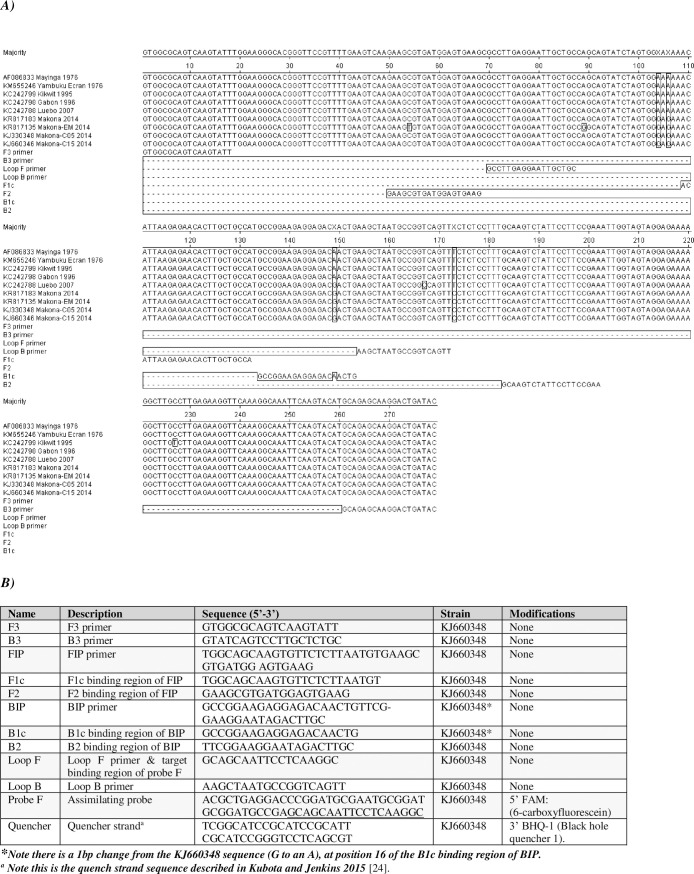
Assay design. ***(A) Alignment of Ebola virus strains at the assay design region*.** Alignment of a selection of strains representing the genetic diversity of Ebola virus, with inclusion of the LAMP primer sequences and target binding regions for the FIP (F1c and F2) and BIP (B1c and B2) primers and assimilating probe. (***B) Primer and probe sequences*.** Table showing primer and probe sequences and individual strand target binding regions of the FIP and BIP primers and Probe F.

The LAMP assay was developed by preparing alignments of the Ebola virus NP coding region to detect sections with good sequence conservation. A small number of single nucleotide changes were apparent for some of the LAMP primers (B1c, F2 and Loop B sequences) (Fig 1A); this was unavoidable due to the natural variability of the Ebola virus genome. A series of LAMP primer sets were designed across the entire 2220 nucleotide section. Each was tested for detection of synthetic nucleic acid fragments using the DNA integrating dye SYBR Safe and a lead set was chosen (Fig 1B). A BLAST (blast.ncbi.nlm.nih.gov) search of the target region was used to confirm specificity.

The LAMP assay was designed to have three detection methods; a real-time assimilating probe-based detection method suitable for multiplexing (Fig 1B), a standard intercalating dye-based real-time method to provide a basis for comparison and an endpoint colour change detection method [23–25]. There have been several endpoint detection methods described for LAMP assays in the literature including turbidity measurement and several colour change methods. We chose to use the warmstart colour LAMP kit from NEB, a pH indicator dye-based method, based on a combination of its sensitivity and the clarity of the colour change observed (pink to yellow).

### Detection limit

**Fig 2:**
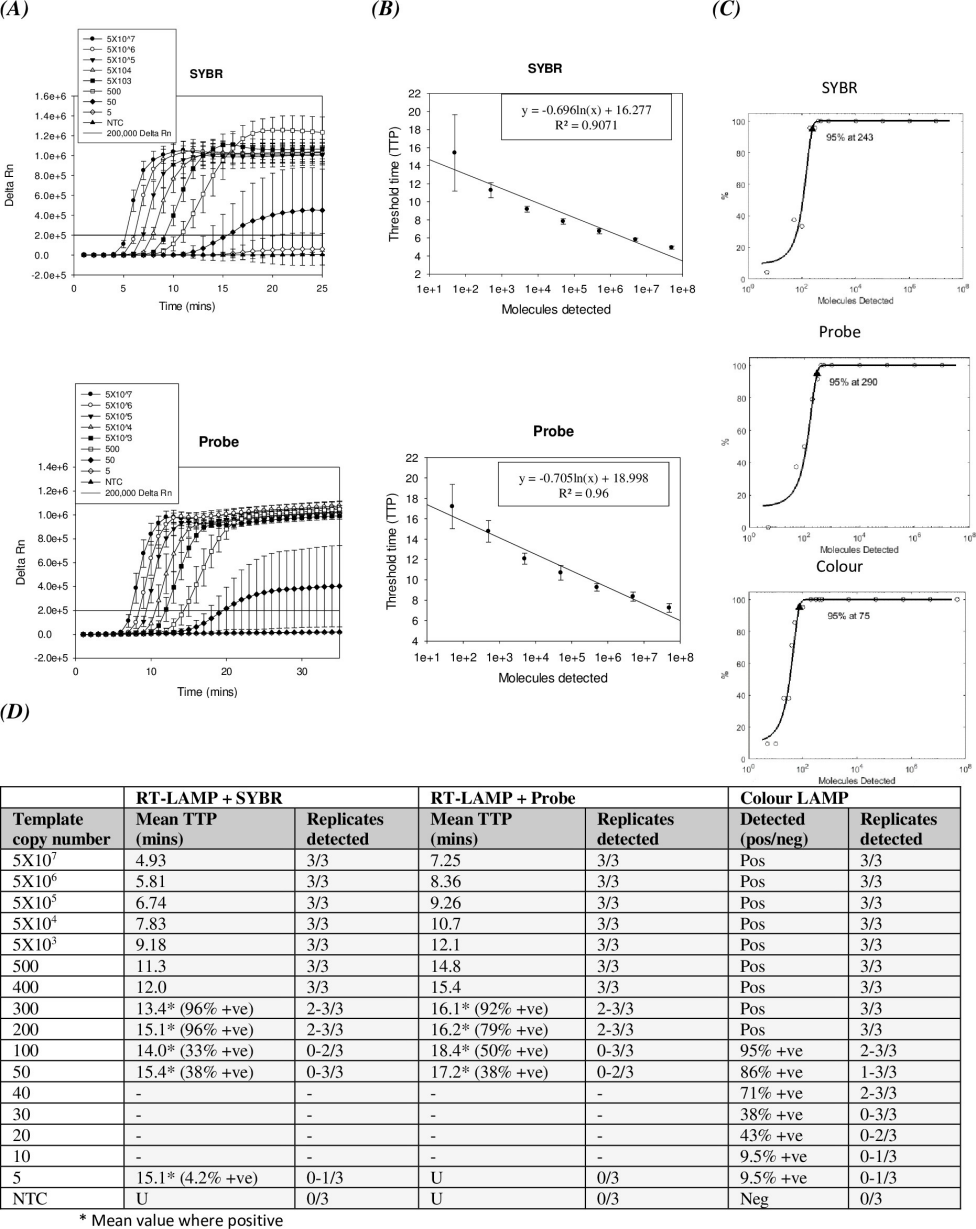
Limit of detection. Limit of detection of the Ebola virus LAMP assays with a serial (1 in 10) dilution of synthetic RNA template of the AY354458 strain. The data is represented graphically as Delta Rn against time (minutes) ***(A)***, graphically as TTP vs copy number ***(B)*,** as a Probit analysis performed using the statistical software Matlab for more accurate detection limit approximation ***(C)*** and in the form of a table of time to positive (TTP value) vs target copy number ***(D)***. Note the values shown are the mean of 8 independent experiments (7 for colour LAMP Probit), each of which were performed with three replicates. The threshold was set at delta Rn 200,000 for the real-time assays.

The limit of detection for the LAMP assay was determined for each of the assay detection outputs (real-time intercalating dye, real-time probe and endpoint colour change) using a serial dilution of a synthetic 2kb RNA fragment of the NP region of a 1995 outbreak strain AY354458 (Fig 2), a representative Ebola virus strain. The data is shown graphically in the form of Delta Rn against time (minutes) (Fig 2A), threshold time (time to positive; TTP) against number of molecules detected (Fig 2B), as a Probit analysis to enable mathematical approximation of the assay detection limit (Fig 2C) and as a table of threshold time and number of replicates detected (Fig 2D). All three detection formats of this assay performed well. Real-time methods detected down to 200 copies of target (96% and 79% respectively for SYBR Safe and probe-based detection), as well as partial detection at 50 copies (38% detection for both) (Fig 2D). The Probit analysis, which calculates the minimum target copy number with 95% detection, by plotting percentage detection against target copy number and fitting to a Probit curve, predicted a limit of detection (LOD) of 243 copies for the intercalating dye assay and 290 for the probe-based assay (Fig 2C). The intercalating dye real-time LAMP assay demonstrated faster time to detection than the probe assay with a 2-3-minute time difference (Fig 2D). It was however necessary to apply a time cut-off for the intercalating dye assay (set at 25 mins) due to the occasional appearance of late false positives, which were absent from both the probe-based detection method and the colourimetric assay. The colourimetric assay proved to be the most sensitive of the three detection formats, with clear detection at 50 copies (86%), partial detection was seen at 20 copies (43% +ve) (Fig 2D) and Probit analysis predicted a LOD of 75 copies/reaction (Fig 2C). For the real-time assays there is good correlation between copy number and time to positive (Fig 2B), suggesting that these are quantitative/semi-quantitative methods.

### Cross-strain detection of Ebola virus

**Fig 3:**
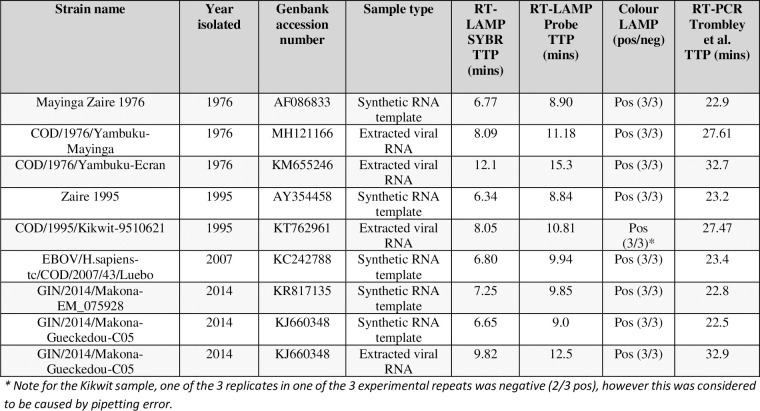
Cross-strain detection of Ebola virus. Table showing detection of Ebola virus extracts and synthetic RNA templates by LAMP assay, in the form of time to positive for the real-time-LAMP assays (TTP; minutes) and positive or negative (pos/neg) for the colour assay, as well as number of replicates detected. The synthetic RNA templates were used at 5X10^6^ copies/reaction and viral extracts were confirmed positive by RT-PCR. The RT-PCR time to positive (TTP) values are included in the table. Note the Ebola LAMP values shown are the mean of 3 independent experiments, each of which were performed with three replicates. The threshold was set at delta Rn 200,000.

The LAMP assays were tested for their ability to identify a panel of Ebola virus strains. The panel represented a cross-section of outbreak strains from 1975–2014 (Fig 3) and included whole genome viral extracts from PHE’s reference collection and 5 synthetic 2kb RNA fragments which were tested at 5X10^6^ copies/reaction. All samples were strongly detected by each of the assay formats, suggesting that the assays have good cross-strain detection (Fig 3). The intercalating dye LAMP assay detected all samples between 6.7 and 12.1 mins and the probe LAMP assay between 8.8 and 15.3 mins. This is consistent with the limit of detection data which also showed more rapid detection (2–3 mins faster) with the intercalating dye assay. When compared to a RT-PCR assay (Trombley et al [27]), which is used by PHE as a gold standard, both real-time-LAMP outputs showed a good time to detection, with consistency across assays, i.e. extracts that showed more rapid detection by RT-PCR also showed more rapid detection in the LAMP assays (Fig 3).

### Negative panel testing

**Fig 4:**
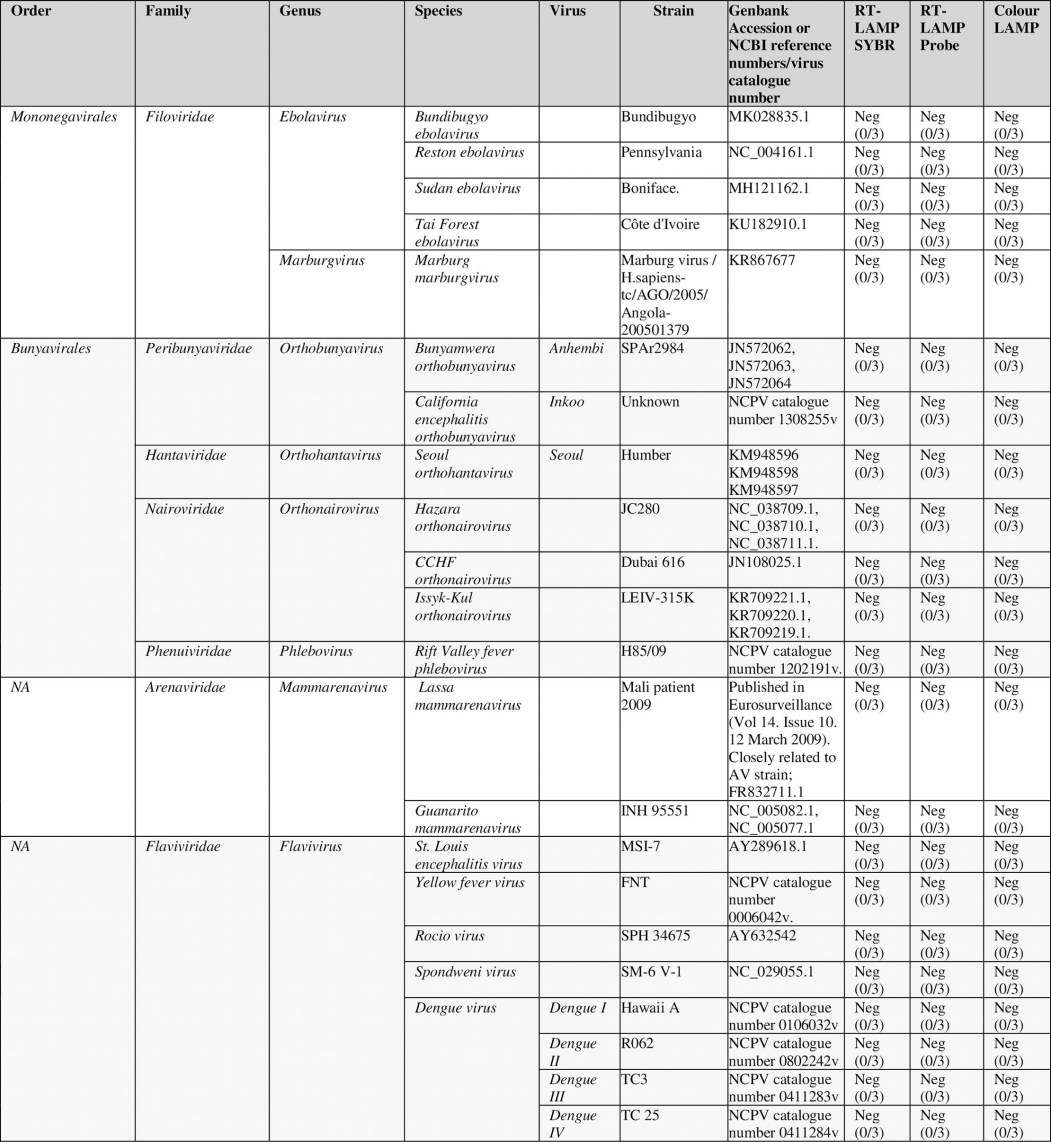
Negative viral panel testing. Table showing the results of a SYBR-LAMP, probe-LAMP and colour-LAMP run with a negative viral panel, consisting of a selection of strains from the *ebolavirus*, *marburgvirus*, *Orthobunyavirus Orthohantavirus*, *Phlebovirus*, *Mammarenavirus*, *Flavivirus* and *Orthonairovirus* genera. Results represent 3 separate experiments, each performed with 3 replicates.

Each of the assay detection formats were tested for potential cross-reactivity with a panel of viral extracts. The panel was selected on the basis of either genetic relatedness to Ebola virus (i.e. non-*Zaire ebolavirus*, *ebolavirus* strains or *marburgvirus*) and other viruses which are the aetiology of similar disease symptoms, supporting the assays utility in differential diagnosis, i.e. VHFs and other fever causing viruses. None of the non-Z*aire ebolavirus ebolaviruses* or the *marburgvirus* were detected using any of the LAMP detection formats, suggesting this is a *Zaire ebolavirus*-specific Ebola assay. None of the VHFs or other fever causing viruses were detected by any of the assay formats, suggesting this assay should be suitable to form the basis of a differential diagnosis for EVD (Fig 4).

### Effect of inhibitors in crude sample preparations

**Fig 5:**
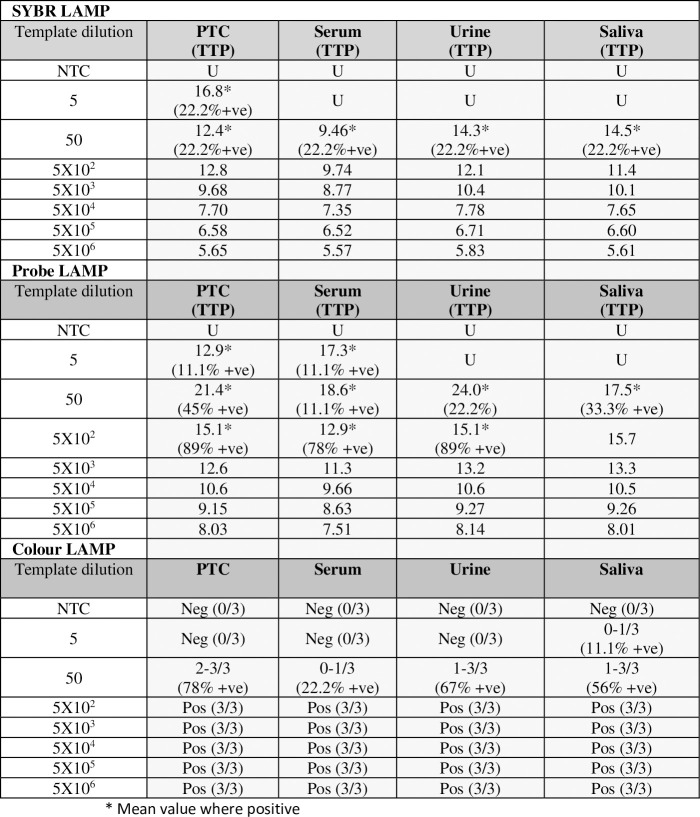
Effect of inhibitors in crude sample preparations on assay sensitivity–limit of detection. Real-time-SYBR, real-time-probe and colour LAMP assays performed with a serial (1 in 10) dilution (from 5X10^6^ copies/reaction) of synthetic RNA template of Ebola virus strain AY354458 and 1 in 10 diluted crude preparations of human serum, saliva and urine. The real-time-LAMP results are shown as time to positive (TTP; minutes), with the threshold set at delta Rn 200,000. The colour LAMP results are shown as positive or negative (pos/neg). All LAMP data represents the mean of 3 independent experiments, each of which were performed with three replicates.

Isothermal molecular diagnostics are considered potentially suitable for testing minimally processed clinical samples, as they may be less susceptible to inhibition due to the presence of contaminants in unextracted bodily fluids. We set out to test this hypothesis by setting up the LAMP reaction in the presence of pooled human bodily fluids with minimal dilution (1 in 10 diluted pools of human serum, saliva and urine from healthy donors). The LAMP reaction was performed with a dilution series of the synthetic Ebola NP fragment AY354458, to enable determination of the effect of contaminants on the detection limit of the assay, with each of the three detection formats (Fig 5). The presence of diluted serum, urine and saliva in the intercalating dye LAMP assay showed little to no inhibitory effect, with full (100%) detection down to 500 copies and partial detection (22% n = 2/9) at 50 copies with and without the contaminants. At 5 copies per reaction only the positive control (PTC) with no contaminants showed any positivity (22% n = 2/9). However, as the Probit analysis predicts a LOD of 243 we would not expect consistent detection below this level and there is no effect on the time to detection. The inhibitory effects of contaminants in crude samples therefore appears to be negligible for the intercalating dye assay The probe-based LAMP format showed near to complete (78–100%) detection at 500 copies of target in the presence and absence of contaminants (PTC 89% (n = 8/9), saliva 100% (n = 9/9), urine 89% (n = 8/9) and serum 78% (n = 7/9)) and partial detection at 50 copies (PTC 45% (n = 4/9), serum 11.1% (n = 1/9) urine 22% (n = 2/9) and saliva 33% (n = 3/9)), though the level of detection varies. Again, some partial detection was apparent for the control and serum sample only at 5 copies. The variable levels of detection at 50 and 5 copies could suggest there is some limited inhibitory effect of the presence of the contaminants, but this is negligible as we would not expect consistent detection below the 290 copies detection limit. There is no effect of the contaminants in the time to positive in the probe-based detection. The colour-detection showed full (100%) detection down to 500 copies for all test conditions and partial detection down to 50 copies (PTC 78% (n = 7/9), serum 22% (n = 2/9), urine 67% (n = 6/9), saliva 56% (n = 5/9)). The reduced percentage detection in the presence of sera suggests a minimal inhibitory effect of unextracted sera on the colour format. It should also be noted that the addition of sera, even diluted 1 in 10 effects the hue of the colour seen in the colour assay in the absence of target Ebola virus RNA (see S3 Fig). The colour in the absence of target should be a bright magenta pink, with a colour change to bright yellow in the event of amplification of target RNA (S3 Fig). When 1 in 10 diluted sera is added in the absence of target, a partial colour change occurs, from magenta pink to an orangey-pink. This does not impact on interpretation of results however, as there is still a clear colourimetric change to bright yellow in the positive samples (S3 Fig).

### Testing of 2013/16 outbreak samples from Sierra Leone

**Fig 6:**
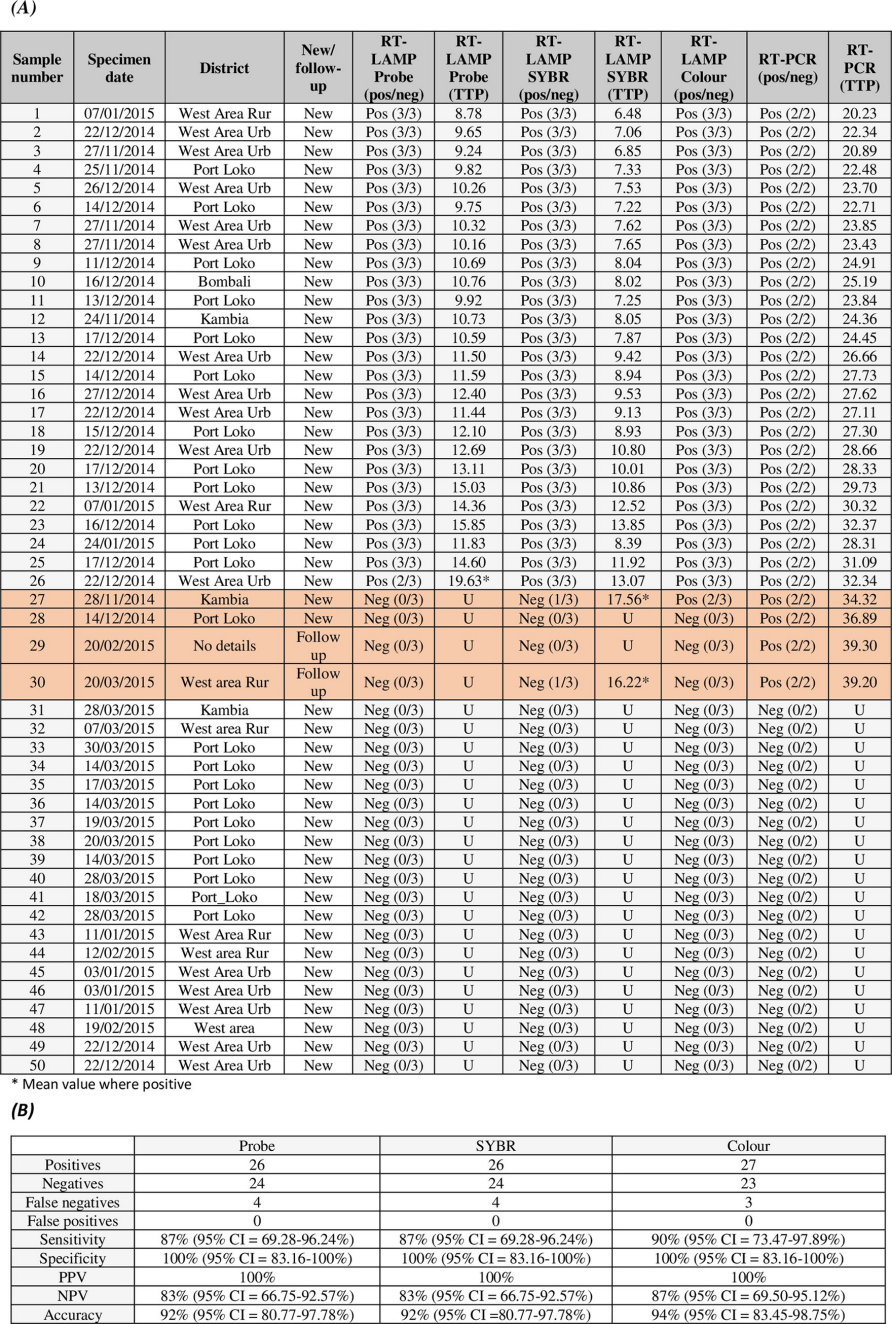
Testing of field samples from the 2013/16 West Africa Ebola virus outbreak. Real-time-SYBR, real-time-probe and colour LAMP testing of a collection of field samples (human sera) from the 2013/16 outbreak in West Africa. (***A)*** The LAMP results are shown as time to positive (TTP; minutes and/or pos/neg) and number of replicates detected. The data represents a single experiment with 3 replicates. The threshold was set at delta Rn 200,000. RT-qPCR data is also included for comparison, with the values shown representing the mean time to positive (TTP; mins). (***B)*** Statistical data including sensitivity, specificity, PPV, NPV and accuracy.

To determine whether the assay in each of its formats is of sufficient sensitivity to detect Ebola virus in real clinical samples and to check for potential false positives, it was necessary to test the LAMP with a collection of clinical Ebola virus outbreak samples. We therefore obtained permission to test samples from the PHE MOHS Ebola Biobank. The biobank is a collection of residual Ebola virus positive and negative serum samples that were tested at PHE laboratories in Sierra Leone during the outbreak in 2013/16 and tested again by PHE in the UK, to confirm sample status and provide information for biobank curators (data shown in far RHS columns of Fig 6). The three LAMP detection formats performed well and produced comparable data with the 50 tested samples. The real-time formats detected 87% of all positive samples (new and follow up samples), and 93% of all new case samples (Fig 6). The colour format detected 90% of all samples (detection of 1 additional positive) and 96% of all non-follow-up samples. No positive results were apparent for any of the negative sera samples for any of the detection formats, suggesting there are no signs of cross-reactivity with non-Ebola virus contaminants in patient sera. As observed in all previous data, the probe detection method had a slightly slower time to positivity, with a delay in detection of between 2 and 3 minutes when compared to the intercalating dye assay (Fig 6).

## Discussion

In this study we set out to develop a new LAMP assay for the specific detection of Ebola virus. We aimed to design a flexible diagnostic suitable for use in the field as a simple to interpret and rapid test appropriate for a low-power portable device with minimal laboratory set-up, but also scalable for a high-throughput setting, where a fast-turnaround is of benefit, with the option to multiplex for expansion of the test’s utility.

The two detection formats we chose to develop were a colourimetric assay for ease of interpretation and a real-time probe-based method to provide more information (e.g. time to positive values), which is also suitable for multiplexing if necessary. A conventional intercalating-dye-based method was also developed to form a basis for comparison.

The LAMP assay was successfully developed and shown to work with the three detection formats. Both real-time assay formats were comparable in limit of detection with a synthetic target, though the intercalating dye assay performed better than the probe assay in time to detection (2–3 minutes faster) (Fig 2). The time to detection for both real-time formats, however, compares favourably with published RT-qPCR assays [27, 34–36] and RT-qPCR data for Ebola virus presented in this paper, with a significant improvement in speed (detection of high copy number target within 5 mins for intercalating dye detection and 7.25 mins for probe detection). All formats showed good detection down to 200 copies of target, with partial results at 50 copies. The colour assay showed additional sensitivity with good detection down to 50 copies and partial results at 20 copies (Fig 2). Although these may not be as sensitive as some already published RT-PCR assays for Ebola virus [36], they nevertheless show detection well within the clinical range [34, 37–39] and are comparable to other published isothermal assays with limits of detection commonly within the range of 50–500 copies of target [26, 40–44] and showing a significant improvement in limit of detection to the recently published Ebola virus RPA [44].

All three formats showed good multi-outbreak cross-Ebola virus sample detection, with recognition of all tested samples, and no cross-reactivity with any non-*Zaire ebolavirus*, *ebolavirus* strains or any related viruses or viruses causing similar symptoms. This demonstrates the utility of this assay as a highly-specific Pan-Ebola virus assay, suitable for the detection of Ebola virus in clinical samples. The probe-based format also has the potential for multiplexing with additional targets to enable the development of a rapid isothermal VHF screen.

When tested with a panel of clinical samples, all the assay formats performed well and with comparable results (87–90% sensitivity, 100% PPV, 83–87% NPV and 92–94% accuracy) and no false positives. The clinical panel was chosen to represent a spread of Ct values to help determine the detection limits of the different assay formats with real clinical samples and although it is difficult to assess how representative the chosen panel is of the spread of Ct values in the entire outbreak, the detection limits suggest it will pick up the majority of acute cases [34, 37–39]. Although the detection rate was not as high as the reference laboratory—gold standard RT-qPCR [27], it represents a good result for a rapid test required for the detection of a high titre pathogen [45]. The benefits of portability, speed, ease-of-use and low infrastructure requirements of the LAMP assays should not be overlooked when assessing potential diagnostic tools. As with any diagnostic there is the possibility of missing some lower titre positives, including pre- or sub- clinical cases [46, 47], or the tail end of disease in recovering patients [38]. These diagnostics will rapidly detect most acute cases however and would greatly enhance the feasibility of rolling out testing capabilities to remote resource-limited settings, where such testing is not normally possible. It would also avoid time delays associated with sample transfer to centralised laboratories, during which time samples might have the opportunity to degrade.

The results of this work suggested these LAMP assay formats are minimally affected on the molecular detection level by the contaminants in crude unprocessed human bodily fluids from healthy individuals. It should however be noted that further studies will be required to fully assess the potential for use of unprocessed clinical samples with these assays. This will involve working with live virus (cultured virus spiked into human bodily fluid samples and/or real clinical samples). The main considerations of this work will be to find a safe and effective method of both inactivating the virus and breaking open the viral envelope to allow access to the genome for testing (taking into account the biosafety considerations of handling a HG4 virus). The additional potential for inhibitory effects of both an inactivating agent and the use of clinical material from unwell patients should also be considered.

The LAMP assays designed in this paper also have the benefit of being an adaptable off-the-shelf solution, open to most research and diagnostic laboratories. All components can be purchased cheaply and readily, and the detection methods require no specialised equipment. While we performed testing on a conventional high-throughput 96 well plate-based thermocycler, the assays can be equally performed on a variety of platforms as demonstrated by our preliminary data (S4 Fig). The platform flexibility for these assays allow them to be tailored to current laboratory and equipment constraints with no large capital investment in most cases. With a reagent cost estimated at £1.80/reaction this should be affordable to most diagnostic laboratories. In a particularly resource-poor setting, where no thermocycler is available and no budget can be found for a relatively cheap portable real-time detector, the colour assay format can be run in the absence of a thermocycler, with only the need for a heat block or water bath to allow the assay to be incubated at a constant temperature, with detection of a simple colour change.

Since the Ebola virus outbreak of 2013–16 there has been an explosion of new candidate molecular diagnostics for Ebola virus in the published literature, with a select few available for purchase or utilised under an Emergency Use Assessment (EUA). Some of these assays are described as being suitable for rapid POC use, but many are based on conventional RT-PCR and are likely to require high specification/reference laboratory infrastructure and higher power for thermal cycling steps, limiting portability of the run platform [14–16]. In contrast, our newly developed real-time LAMP assays may be performed on small, portable, battery powered isothermal real-time detectors [23] such as the OptiGene Genie devices [48, 49]. The developed LAMP assays could also theoretically be run on a fully automated sample-to-answer device, cutting out the need for any technical expertise. A sample-to-answer device for these assays could potentially be scaled down by removing the need for extraction, making it smaller, more lightweight and cheaper to run [50].

Other LAMP-based methods have been designed which show promise as Ebola virus diagnostics, with good detection on real-time devices. These lack the potential to multiplex, however, making the detection of multiple pathogens or the inclusion of internal reference control difficult [40, 51–53]. This versatility is a simple option in our probe-based Ebola LAMP, meaning there is clear potential to use this method as the real-time LAMP-based diagnostic of choice during an Ebola virus outbreak. There are other types of isothermal assays for Ebola virus published in the literature [44], however these have a higher potential for amplifying a non-target product as they lack the complex mix of different primers in a LAMP assay [22]. LAMP also has adaptability in the potential detection methods it employs, with endpoint fluorescence, colour detection and turbidity measures all providing alternatives in the absence of a real-time device [41, 42, 54, 55], which could be of benefit in the field where the high specification/reference laboratory infrastructure, power needs and resources could be limiting. Other types of isothermal amplification assay, which use paper-based microfluidics in a simplified set-up and results interpretation format, have also been described for the detection of Ebola virus [56, 57]. Whilst retaining the potential for multiplexing (albeit as individual parallel reactions) however, some difficulties relating to lack of sensitivity and stability, suggest such methods need further development [56, 57]. In addition, there is also the need for custom equipment. No such issues exist for the LAMP assays described in this paper.

There are many examples in the literature of endpoint LAMP assays with visual detection, such as detection of the pyrophosphate biproduct of the amplification by turbidity or through colour change using a calcein dye, and detection of Mg^2+^ ions with hydroxy napthol blue (HNB) dye, however, the results with these assays have been mixed [41, 42, 54]. Turbidity detection assays tend to have limited sensitivity with “by-eye” detection. Some papers describe use of a turbidometer for lower copy number observation, which adds complexity and increases equipment need, a problem which also exists for nucleic acid strip based visual detection methods [58]. The existing colour assays are often difficult to interpret, due to the minimal difference between the colour change detected, making it problematic to interpret by eye. Some publications only show data visualised in the presence of a UV source, which again adds to the complexity and equipment needs [59, 60]. The colour change LAMP assay included in this paper is very easy to interpret, with a clear change from a bright magenta pink, to a bright yellow which simplifies interpretation for operators with minimal training.

With the advent of whole genome sequencing, the role of simple nucleic acid amplification test (NAAT)-based diagnostics in the future is sometimes questioned. These assays however serve a very different need; simple sample-to-answer NAATs are cheap, quick and straightforward diagnostic methods. They are vital in outbreak situations where large numbers of samples need to be tested rapidly, in resource limited settings. Whole genome sequencing on the other hand is a more expensive and time-consuming method. While it can offer data to support contact tracing and potential new transmission patterns, it requires intensive laboratory back up and is currently more suited to outbreak follow up.

Overall this paper describes the successful development of a new set of rapid diagnostics for Ebola virus, which are suited to field use. This could be of vital importance for the next EVD outbreak, bringing diagnostics closer to the communities where they are needed and expediting patient triage.

## Supporting information

S1 Fig Information table for synthetic Ebola virus NP gene DNA fragments with SP6 and T7 promoters. (TIF)

S2 Fig Sequences of synthetic Ebola virus NP gene DNA fragments with SP6 and T7 promoters. Shown are the approx. 2kb DNA fragments (5’-3’ orientation) designed to be a template for *in vitro* transcription to create the synthetic RNA templates used to test the LAMP assays. The fragments are composed of a section of the NP gene of Ebola virus (in black), a T7 promoter (20bp in blue) and an SP6 promoter (20bp in green), flanked by GC-rich tails (4bp). ***(A) AF086833 NP gene DNA fragment (1*.*988 kb*, *including 1940bp coding region)*. *(B) AY354458 NP gene DNA fragment (1*.*988 kb*, *including 1940bp coding region) (C) KC242788 NP gene DNA fragment (1*.*988 kb*, *including 1940bp coding region*.*) (D) KR817135 NP gene DNA fragment (1*.*988 kb*, *including 1940bp coding region)*. *(E) KJ660348 NP gene DNA fragment (2kb*, *including 1952bp coding region)***
(TIF)

S3 Fig Colour change in the colour Ebola virus LAMP in the presence and absence of 1 in 10 diluted pooled human sera. Shown is a serial (1 in 10) dilution of synthetic RNA template of the AY354458 strain in the presence and absence of 1 in 10 diluted pooled human serum.
(TIF)

S4 Fig Initial testing on a portable real-time device (OptiGene Genie III). Real-time data with the integrating dye assay (AF086833 at 5X10^6^-5 copies/reaction and no template control)
(TIF)

S5 Fig Checklist: STARD checklist. (DOCX)
