# Supplementary figures and images for: A flexible format LAMP assay for rapid detection of Ebola virus
Source: PLoS Negl Trop Dis. 2020 Jul 31;14(7):e0008496. doi: 10.1371/journal.pntd.0008496 (PMC7423149; doi:10.1371/journal.pntd.0008496)

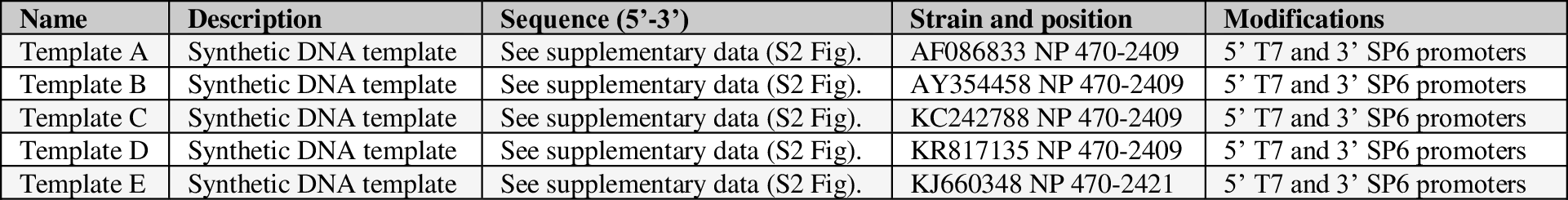

Supplement: S1 Fig — (TIF) [file pntd.0008496.s001.tif]

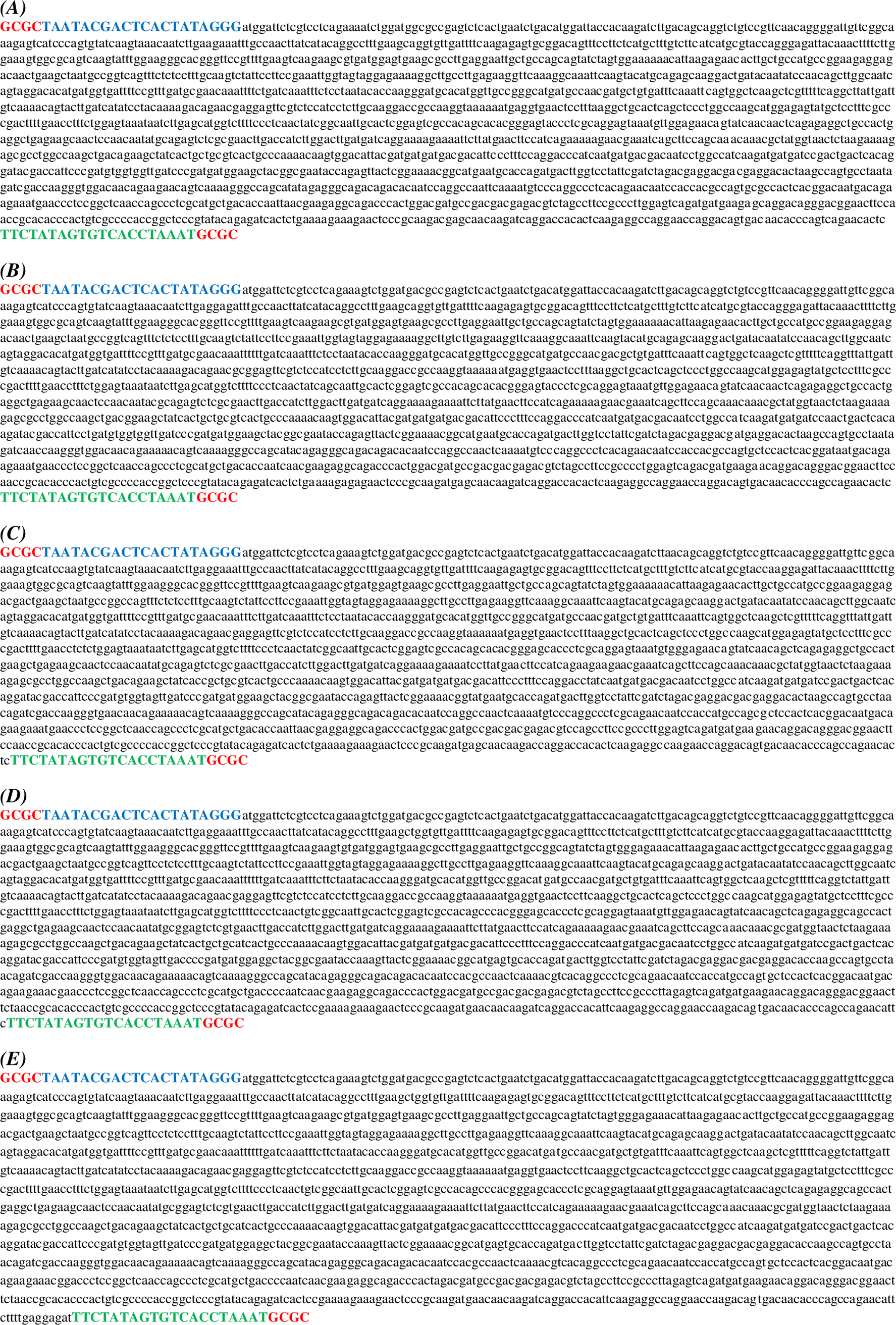

Supplement: S2 Fig — Shown are the approx. 2kb DNA fragments (5’-3’ orientation) designed to be a template for in vitro transcription to create the synthetic RNA templates used to test the LAMP assays. The fragments are composed of a section of the NP gene of Ebola virus (in black), a T7 promoter (20bp in blue) and an SP6 promoter (20bp in green), flanked by GC-rich tails (4bp). (A) AF086833 NP gene DNA fragment (1.988 kb, including 1940bp coding region). (B) AY354458 NP gene DNA fragment (1.988 kb, including 1940bp coding region) (C) KC242788 NP gene DNA fragment (1.988 kb, including 1940bp coding region.) (D) KR817135 NP gene DNA fragment (1.988 kb, including 1940bp coding region). (E) KJ660348 NP gene DNA fragment (2kb, including 1952bp coding region) (TIF) [file pntd.0008496.s002.tif]

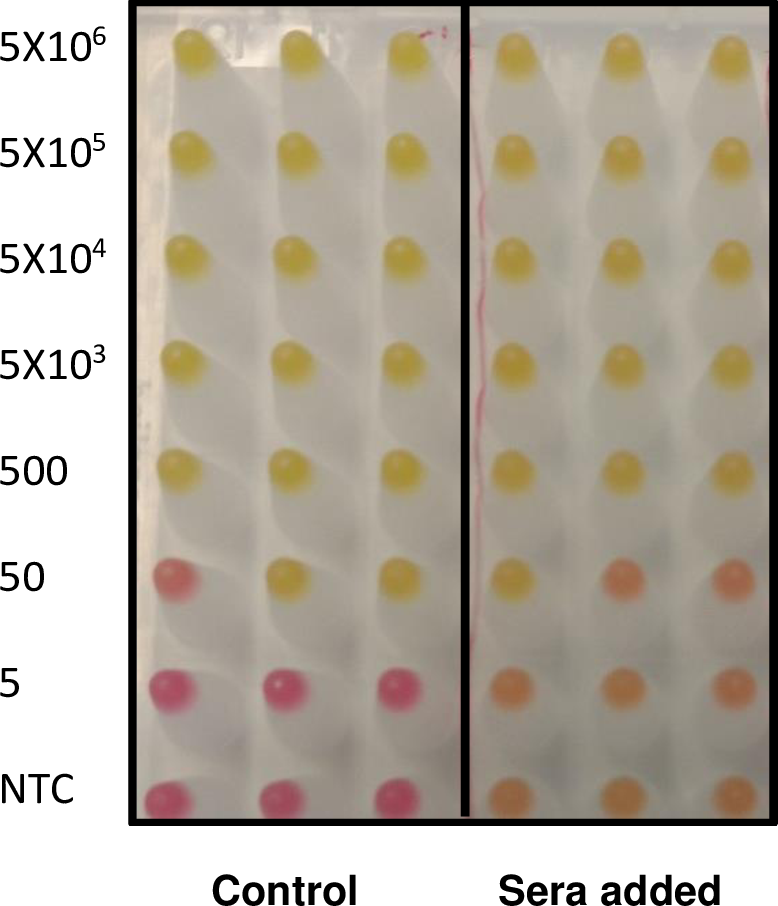

Supplement: S3 Fig — Shown is a serial (1 in 10) dilution of synthetic RNA template of the AY354458 strain in the presence and absence of 1 in 10 diluted pooled human serum. (TIF) [file pntd.0008496.s003.tif]

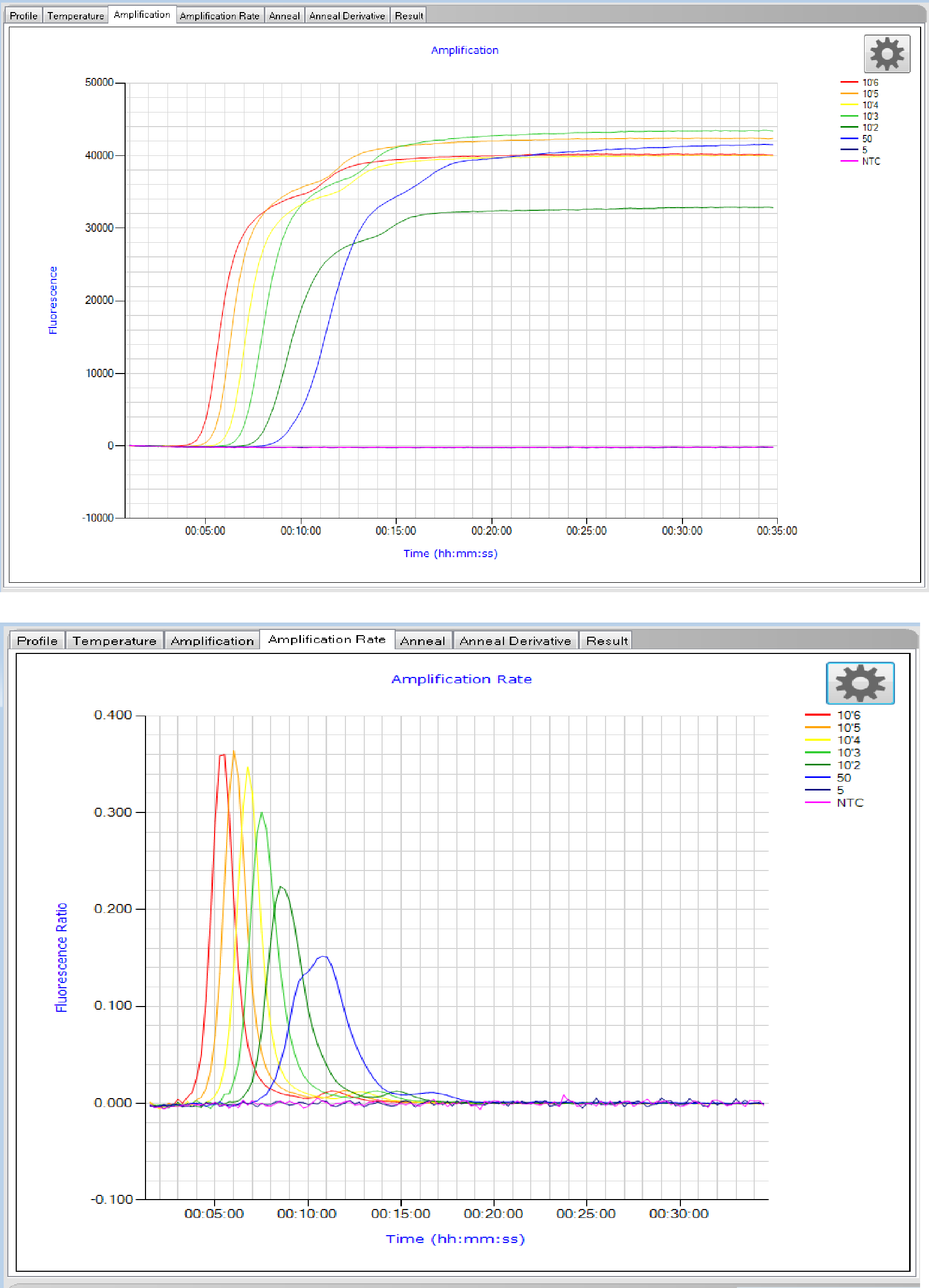

Supplement: S4 Fig — Real-time data with the integrating dye assay (AF086833 at 5X106-5 copies/reaction and no template control) (TIF) [file pntd.0008496.s004.tif]
